# Immune-Related lncRNA Correlated with Transcription Factors Provide Strong Prognostic Prediction in Gliomas

**DOI:** 10.1155/2020/2319194

**Published:** 2020-10-30

**Authors:** Yixin Tian, Yi-Quan Ke, Yanxia Ma

**Affiliations:** ^1^Department of Neurosurgery, Zhujiang Hospital, Southern Medical University, Guangzhou, China; ^2^The National Key Clinical Specialty, The Engineering Technology Research Center of Education Ministry of China, Guangdong Provincial Key Laboratory on Brain Function Repair and Regeneration, Guangzhou, China

## Abstract

Glioma is the most common and deadly tumor in central nervous system. According to previous studies, long noncoding RNAs (lncRNA) and transcription factors were significant factors of gliomas progression by regulating gliomas immune microenvironment. In our study, we built two independent cohorts from CGGA and TCGA. And we extracted 253 immune-related lncRNA correlated with prognosis. After LASSO analysis and multivariate Cox regression analysis, 8 immune-related lncRNA were used to construct classifier. The effectiveness of classifier was confirmed in both CGGA (AUC = 0.869) and TCGA (AUC = 0.902) cohorts. The correlation between transcription factors and immune-related lncRNA was calculated by WCGNA. Eventually, we built a network between 8 lncRNA and transcription factors. The function of core immune-related lncRNA in gliomas immune microenvironment was also investigated by CIBERTSORT. Our research provided a strong classifier of immune-related lncRNA to predict gliomas patient outcome. We also found the correlation between core immune-related lncRNA and transcription factors. These results may stimulate new strategy of immunotherapy in gliomas patients.

## 1. Introduction

Glioma is the most aggressive tumor in central nervous system and patients with gliomas have poor prognosis. As the 2016 WHO classification of central nervous system tumors, immunoreactivity and molecular alterations were considered as classifiers. For example, Glioblastomas have been classified by IDH immunoreactivity as IDH-mutant, IDH-wildtype, and IDH-NOS. Oligodendroglioma diagnosis now requires an identified mutation of IDH and a codeletion of 1p19q. Thus, discovery of effective prognostic biomarkers was significant towards gliomas patients. Tumor microenvironment (TME) was illuminated as a significant player of gliomas progression, metastasis, and recurrence [1]. Recent studies have demonstrated the function of different types of immune cells in the glioma microenvironment [2, 3]. For example, M2 macrophages promoted gliomas progression while M1 macrophages played the antitumor role [4]. In addition, the discovery of molecular function of immune cells may stimulate new strategies of immunotherapy in gliomas patients [5].

Long noncoding RNAs (lncRNA) played significant role in plenty of biological processes such as regulation of transcription, translation, and protein modification [6]. And previous research studies revealed that lncRNA presented strong power of prognosis prediction in many cancer types [7, 8]. For example, GAS5 was an independent molecular cancer biomarker in bladder cancer patients [9], and a cell cycle-associated lncRNA, HOXA11-AS, was a biomarker of progression in glioma [10]. Several researches revealed the pro/antitumor function of lncRNA in different types of cancers by regulated immune responses. Neftel et al. found that lncRNA Sros1 promoted IFN-*γ*-mediated activation of innate immune responses by stabilizing Stat1 mRNA [11], and Zhao et al. revealed that LncRNA SNHG14/miR-5590-3p/ZEB1 positive feedback loop promoted diffuse large B cell lymphoma progression through regulating PD-1/PD-L1 checkpoint [12]. Therefore, the identification of immune-related lncRNA provided possible mechanism in gliomas microenvironment. In addition, transcription factors affect different immune cell types that are important in the tumor progression. Atsaves V et al. found the roles of AP-1 in the regulation of antitumor immune responses and provided a new sight of immune checkpoint blockade therapy [13]. However, the relation between immune-related lncRNA and transcription factors in gliomas microenvironment was unknown.

In this study, we built two independent cohorts from CGGA and TCGA. We also extracted immune-related lncRNA correlated with prognosis. After LASSO analysis and multivariate Cox regression analysis, 8 immune-related lncRNA were used to construct classifier. The effectiveness of classifier was confirmed in both CGGA and TCGA cohorts. Eventually, we built a network between 8 lncRNA and transcription factors. The function of core immune-related lncRNA in gliomas immune microenvironment was also investigated by CIBERTSORT. Our research provided a strong classifier of immune-related lncRNA to predict gliomas patient outcome. We also found the correlation between core immune-related lncRNA and transcription factors. These results may stimulate new strategy of immunotherapy in gliomas patients.

## 2. Methods

### 2.1. Collection of RNA-Seq and Matched Clinical Data from CGGA and TCGA

We built two independent cohorts for training set and testing set from CGGA and TCGA. The gene expression profiles were downloaded from the Chinese Gliomas Genome Atlas (CGGA http://www.cgga.org.cn/). 695 RNA-seq data of Chinese gliomas patients were obtained from CGGA database. Moreover, a series of measures were taken: (1) Genes with a variance of 0 were filtered out. (2) Complete follow-up information and samples with a follow-up time >30 days were served. Finally, 464 samples meeting the inclusion criteria were included. Each patient matched specific clinical data, which includes histology, WHO grades, age, gender, chemotherapy, radiotherapy, survival status, and survival duration in days. To build the testing cohort, the RNA-seq FPKM of gliomas including corresponding outcome data were downloaded from the Cancer Genome Atlas (TGCA). The following measures were taken: (1) the IDs were annotated on the basis of hg38 reference genome. (2) Genes with a variance of 0 were filtered out. (3) For the same gene corresponding to multiple IDs or a patient with multiple tumor samples, the average was taken. (4) Samples with a follow-up time >30 days were kept. Eventually, a total of 629 TCGA samples fulfilled our criteria.

### 2.2. Identification of Immune-Related lncRNA

For extracting immune-related lncRNA, we downloaded immune-related gene set which includes immune response and immune system process from the MSigDB. The correlation analysis between lncRNA and immune-related genes was performed by *limma* package. Correlation coefficient >0.4 and *P* value <0.001 were included. Finally, we generated 543 immune-related lncRNA for further analysis.

### 2.3. Generation and Assessment of Prognostic Classifier by Multiple Assays

To select prognostic immune-related lncRNA, univariate Cox regression analysis of continuous variables was performed by survival package. 253 prognostic immune-related lncRNA (*P* < 0.01) were generated for next analysis. Then, the Least Absolute Shrinkage and Selection Operator (LASSO) regression was performed to identify prognostic biomarkers and we set the random seed to 5 before the LASSO analysis. To generate and optimize the prognostic classifier, the multivariate Cox regression analysis was performed. The receiver operator characteristics (ROC) curves were drawn by *survivalROC* package. The area under the curve (AUC) of the ROC curve was calculated and compared to examine the performance of the classifier in both training and testing cohorts. The median risk score (RS) was determined to separate the genes into high-risk and low-risk groups. KM curves and independent test were performed by *survival* package to assess the effectiveness of classifier in gliomas patients.

### 2.4. Functional Enrichment Analysis of High-/Low-Risk Score Groups

To assess the different functions between high- and low-risk groups, GO categories include biological processes (BP), molecular functions (MF), or cellular components (CC), and KEGG (Kyoto Encyclopedia of Genes and Genomes) pathways were analyzed by GSEA (Gene Set Enrichment Analysis). FDR <0.1 and *P* < 0.01 were considered significant.

### 2.5. Generation of Immune-Related lncRNA and Transcription Factors Network

The transcription factors were identified by Cistrome Cancer database, and the expression data of 312 transcription factors were extracted for weighted gene coexpression network analysis (WGCNA). The risk score, clinical information, and expression modules were calculated in WGCNA. A soft-thresholding parameter of 5 was chosen to guarantee a scale-free network. Module eigengenes (MEs) were considered as the most principal component of each gene module and adopted as the representative of all genes in each module. The interesting module was identified by calculating the relevance between MEs and risk score. Gene significance (GS) represented the degree of linear correlation between gene expression of module and risk score. Besides, the average GS for all genes in the module was defined as the module significance (MS). The module with the highest MS score was chosen as the one related to risk score. Eventually, the network between prognostic immune-related lncRNA and chosen transcription factors was built by Cytoscape. In addition, the functional enrichment analysis was performed by *clusterProfiler* package.

### 2.6. Comparison of Gliomas-Infiltrating Immune Cells between High and Low Expressions of Hub lncRNA

We assessed the proportions of 22 immune cell subtypes based on mRNA genes by CIBERSORT package. The perm equal to 1000 and *P* value <0.01 in CIBERSORT analysis result were contained for further analysis. *Pheatmap* package, *corrplot* package, and *vioplot* package were used for plotting.

### 2.7. Statistical Analysis

All analyses were carried out by *R* version 3.6.1 and corresponding packages.

## 3. Result

### 3.1. Construction of Prognostic Immune-Related lncRNA Classifier by Multiple Assays

**Figure 1:**
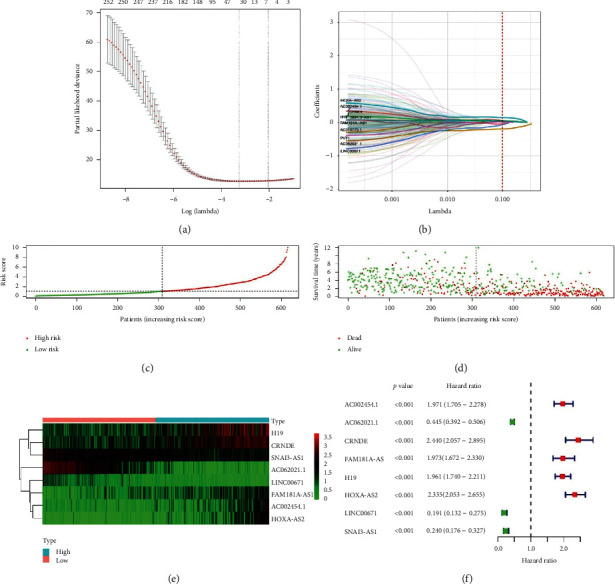
Construction of prognostic immune-related lncRNA classifier. (a, b) The selection of prognostic immune-related lncRNA by LASSO analysis. (c) The distribution of risk score. (d) The survival time and status of patients. (e) The heatmap of selected 8 immune-related lncRNA of the classifier. (f) The univariate Cox regression analysis of 8 immune-related lncRNA.

After screening data, a total of 464 samples from CGGA fulfilled our criteria. We extracted expression data of lncRNA and performed correlation analysis between lncRNA and immune-related genes. As a result, 543 immune-related lncRNA were generated for further analysis (correlation coefficient >0.4 and *P* value <0.001 were included). Next, 543 immune-related lncRNA were filtrated by univariate Cox regression analysis, and 253 prognostic immune-related lncRNA (*P* < 0.01) were generated. To construct classifier, these prognostic immune-related lncRNA were selected for LASSO analysis and optimized by multivariate Cox regression analysis (Figures 1(a) and 1(b)). Eventually, 8 immune-related lncRNA were selected to construct classifier which includes AC002454.1, AC062021.1, CRNDE, FAM181A-AS1, H19, HOXA-AS2, LINC00671, and SNAI3-AS1 (Figures 1(c)–1(f)). We also calculated the risk score (RS) of each sample based on the relative expression level and the corresponding LASSO coefficients in CGGA cohort.

### 3.2. Assessment of the Classifier Efficiency and Verification of the Classifier in TCGA Cohort

**Figure 2:**
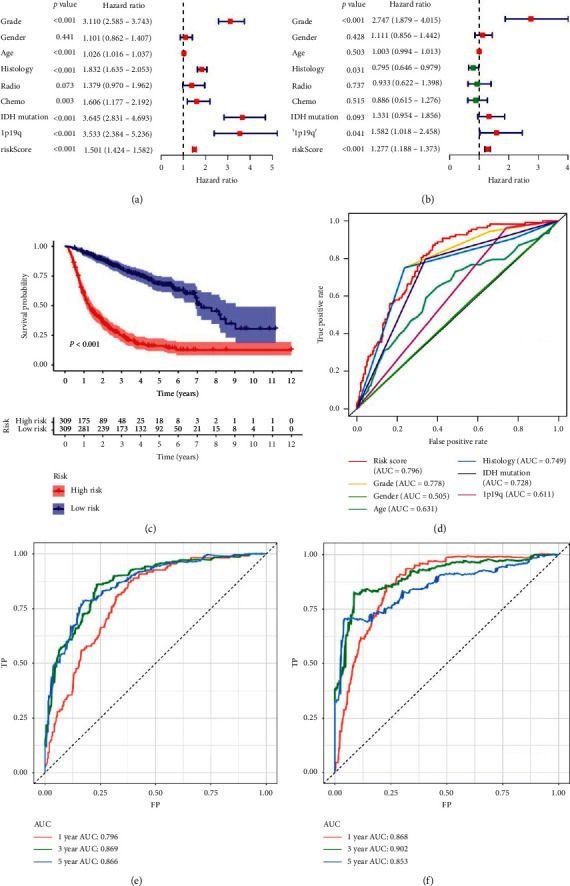
Assessment of the classifier efficiency and verification of the classifier in TCGA cohort. (a) The univariate Cox regression analysis of risk score and several clinical features in gliomas. (b) The multivariate Cox regression analysis of risk score and clinical features. (c) K–M curves of high- and low-risk score groups in CGGA cohort. (d) Multiple ROC curves of risk score and clinical features in CGGA cohort. (e) Time-dependent ROC curves of 1, 3, and 5 years in CGGA cohort. (f) Time-dependent ROC curves of 1, 3, and 5 years in TCGA cohort.

We generated univariate and multivariate analysis which enrolled clinical features and risk score in the overall set. The result showed that the classifier was an independent factor for gliomas patients (Figures 2(a) and 2(b). KM curves showed that the patients with high-risk score had lower survival rate than patients with low-risk score (*P* < 0.001) (Figure 2(c)). To assess the efficiency of the classifier, time-dependent and multiple ROC curves were performed in CGGA cohort. As a result, the AUC was 0.796 in 1 year, 0.869 in 3 years, and 0.866 in 5 years (Figure 2(e)). In addition, the classifier had better predictive power than other clinical features which includes WHO grades, histology, IDH mutation, and 1p19q codeletion (Figure 2(d)). To verify the results, time-dependent ROC curves were performed in TCGA cohort. The AUC was 0.868 in 1 year, 0.902 in 3 years, and 0.853 in 5 years. The results showed the classifier also had favorable accuracy in the validation set (Figure 2(f)).

### 3.3. Clinical Correlation and Functional Enrichment Analysis of High-Risk Score Group

**Figure 3:**
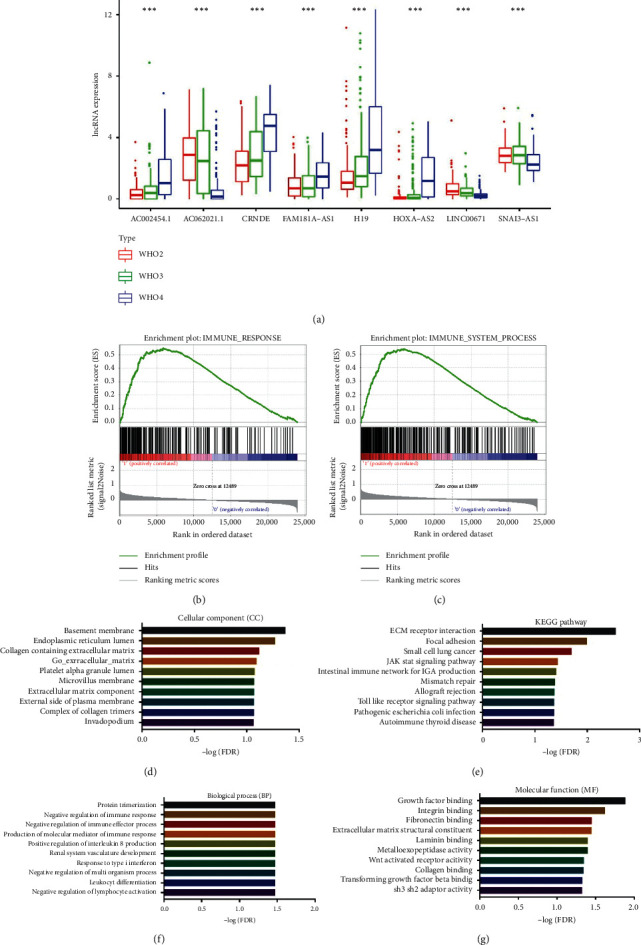
Clinical correlation and functional enrichment analysis of high-risk score group. (a) The correlation between 8 immune-related lncRNA and WHO grades. (b, c) High-risk score group was correlated with immune response and immune system process gene sets by GSEA analysis. (f, d, g, e) Functional enrichment of high-risk score groups in biological process, cellular components, molecular function, and KEGG pathway.

Clinical correlation analysis showed that each of the 8 lncRNA was correlated with WHO grades in gliomas (*P* < 0.001) (Figure 3(a)). Then, we confirmed that high-risk score group was correlated with immune-related gene sets by GSEA analysis (Figures 3(b) and 3(c)). To explore the potential mechanism in high-risk group, functional enrichment analysis was performed by GSEA (Gene Set Enrichment Analysis) (Figures 3(e)–3(g)).

### 3.4. Construction of Network between 8 Immune-Related lncRNA and Transcription Factors

**Figure 4:**
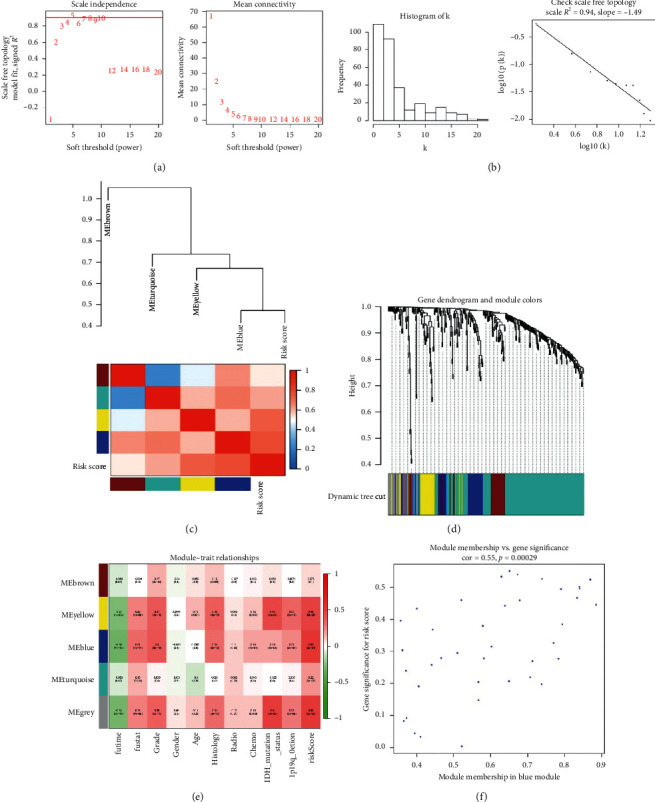
WGCNA of 312 transcription factors in CGGA cohort. (a, b) The scale-free fit index for soft-thresholding powers. (c, d) Dendrogram of all differentially expressed transcription factors clustered based on different metrics. (e) Heatmap of the correlation between module eigengenes and clinical traits. (f) The correlation between gene expression and risk score in blue module.

**Figure 5:**
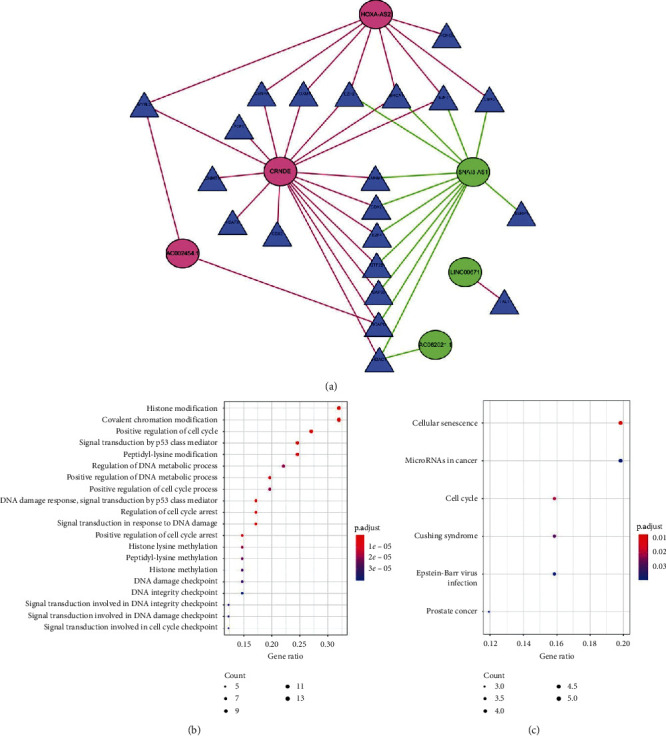
(a) The network between 8 immune-related lncRNA and transcription factors. Circles represent lncRNA, triangles represent transcription factors, red lines mean positive correlations, and green lines mean negative correlations. (b, c) Functional enrichment of selected lncRNA and transcription factors in GO and KEGG.

Based on Cistrome Cancer database, we extracted expression data of 312 transcription factors in CGGA cohort for WGCNA. The soft-thresholding power in the WGCNA was determined based on scale-free R2 (*R*2=0.95). Five modules were identified by the average linkage hierarchical clustering based on the soft-thresholding power. The blue module had the highest correlation with the risk score and contained 40 transcription factors (Figure 4). Then, the correlation analysis between 8 immune-related lncRNA and 40 transcription factors was performed. Then, we constructed the network by Cytoscape. The result showed that 3 high-risk immune-related lncRNA (CRNDE, HOXA-AS2, and AC002454.1) and 3 low-risk immune-related lncRNA (SNAI3-AS1, AC062021.1, and LINC00671) were correlated with transcription factors expression. We also found that CRNDE, HOXA-AS2, and SNAI3-AS1 played core roles in the network (Figure 5(a)). The functional enrichment analysis was performed to explore the potential mechanism (Figures 5(b) and 5(c)).

### 3.5. The Function of Core Immune-Related lncRNA in Gliomas Microenvironment

**Figure 6:**
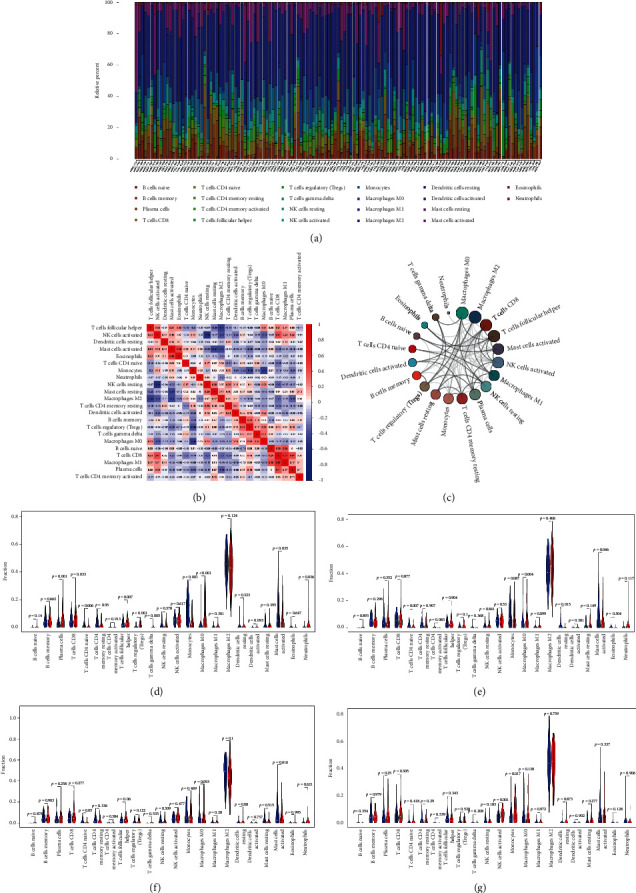
(a) 22 subtypes of immune cells in CGGA cohort. (b, c) Correlation analysis between 22 subtypes of immune cells. (d) The different infiltrating immune cells in high/low-risk score group. (e–g) The different infiltrating immune cells in high/low expression of 3 core lncRNA.

Based on CIBERSORT algorithm, we performed the landscape of tumor infiltrating immune cell subtypes in CGGA cohort (Figures 6(a)–6(c)). Besides, the difference of immune cell subtypes between high-risk and low-risk groups was analyzed. To investigate the function of 3 core immune-related lncRNA in immune system, the median expression was determined to separate the samples into the high-expression and low-expression groups. The results indicated that multiple immune cells showed different infiltration between high-risk and low-risk groups. Plasma cells, CD8 T cells, T cells follicular helper, Tregs, T cells gamma delta, macrophages M0, and neutrophils were significantly increased in high-risk groups, while T cells CD4 naïve, T cells CD4 memory resting, monocytes, and activated mast cells were significantly decreased. Furthermore, the different glioma-infiltrating immune cells between high and low expression of core lncRNA were also calculated (Figures 6(d)–6(g)).

## 4. Discussion

Because of the limitations of the immune response in the central nervous system, there is no successful immunotherapy for gliomas patients currently [14, 15]. Although there have been few failures of immune therapy clinical trials in gliomas, a plenty of different immunotherapies studies are currently being performed in gliomas patients which include immune-checkpoint blockade, CAR T cell therapy, oncolytic viral therapy, and vaccination therapy [16, 17]. Thus, the new strategies of immunotherapy needed to be found in gliomas. Previous studies suggested that lncRNA play a significant role in gliomas progression by regulating gliomas immune microenvironment. For example, Li et al. found that modulating lncRNA SNHG15/CDK6/miR-627 circuit reduces M2-polarization in glioblastoma [18]. Due to the wide expression in multiple tissues and cell lines, lncRNA was also considered as a favorable prognostic biomarker [19]. Besides, increasing evidence suggested that the transcription of lncRNA genes is regulated by multiple core transcription factors applied to protein-coding genes [20, 21]. Therefore, immune-related lncRNA regulated by transcription factors may become novel prognostic biomarkers in gliomas.

In our study, we generated two independent gliomas cohorts in CGGA and TCGA, and we extracted 253 immune-related lncRNA correlated with prognosis. After LASSO analysis and multivariate Cox regression analysis, 8 immune-related lncRNA were used to construct classifier. The classifier showed strong predictive ability in both CGGA and TCGA cohorts. In addition, the classifier had better predictive power than other clinical features which include WHO grades, histology, IDH mutation, and 1p19q codeletion. According to previous studies, lncRNA H19 promoted gliomas progression and angiogenesis by regulating several microRNAs [22–24]. LncRNA HOXA-AS2 regulated malignant glioma behaviors via the RND3 expression and miR-373/EGFR axis [25, 26]. CRNDE promoted malignant progression of glioma by STAT3 and EGFR [27, 28]. In addition, other 5 lncRNA have not been researched in gliomas. Eventually, we built a network between 8 lncRNA and transcription factors. The result shows that CRNDE, HOXA-AS2, and SNAI3-AS1 played core roles in the network. 3 core transcription factors (EZH2, BRCA1, and E2F7) were positively correlated with CRNDE and HOXA-AS2 but negatively correlated with SNAI3-AS1. 6 transcription factors were consistent with CRNDE and HOXA-AS2. 7 transcription factors were positively correlated with CRNDE but negatively with SNAI3-AS1. Besides, 4 transcription factors were positively correlated with HOXA-AS2 but negatively with SNAI3-AS1. In addition, CRNDE and HOXA-AS2 were significantly increased in high grade gliomas while SNAI3-AS1 was deceased.

To investigate the role of classifier and 3 core lncRNA in glioma immune microenvironment, we calculated 22 subtypes of glioma-infiltrating immune cells based on CIBERTSORT [29]. Tregs are correlated with unfavorable prognosis in several kinds of tumor microenvironment [30–32] (e.g., ovarian cancer, breast cancer, kidney cancer, and pancreatic cancer). Our results showed that Tregs were significantly increased in high-risk score group, which demonstrated that Tregs may promote the development of gliomas. Mast cells play an important role in the growth of tumor [33]. However, the contribution of mast cells to the microenvironment of solid malignancies remains controversial. Previous studies have illustrated that gliomas are associated with a profound accumulation of mast cells. Our results showed that activated mast cells were significantly decreased in high-risk group. Consistent with previous study, gliomas may block resting mast cell activation. We found that macrophages M0 were significantly increased in high-risk group while monocytes were decreased. In addition, high expression of CRNDE and HOXA-AS2 showed the same trend while SNAI3-AS1 had opposite trend. These results indicate that the high-risk immune lncRNA (CRNDE and HOXA-AS2) may promote gliomas progression by transforming monocytes into macrophages while the low-risk immune lncRNA conversely inhibit macrophages infiltration. Natural killer (NK) cells are cellular components of the immune system that are more difficult to deceive by tumor cells and have greater cytotoxic activity [34]. Previous studies have found that lncRNA plays an important role in NK cell development and function [35]. Although gliomas are frequently infiltrated by natural killer (NK) cells, these are actively suppressed by the gliomas microenvironment [36, 37]. According to our results, activated NK cells showed no difference or slightly decreased in high expression of CRNDE and HOXA-AS2, but activated NK cells were significantly increased in high expression of SNAI3-AS1. This result suggested that high expression of SNAI3-AS1 may suppress gliomas progression by activating NK cells and be regulated by multiple transcription factors.

However, there were several limitations in our research. First, the calculation results from public database may show bias. Although we have verified the results in two independent cohorts, we should carry out deeper research. Second, the correlation between 3 core lncRNA and transcription factors in gliomas needs further confirmation *in vitro* and *in vivo*.

## 5. Conclusion

We successfully constructed a prognostic classifier based on immune-related lncRNA in gliomas. 8 selected immune-correlated lncRNA were independent prognostic factors and were significantly correlated with WHO grades. We also built a correlation network between immune-related lncRNA and transcription factors. The landscape of gliomas immune microenvironment and the function of core immune-related lncRNA were investigated by CIBERSORT. Our research provided a strong classifier to predict gliomas patient outcome, and these results may stimulate new strategy of immunotherapy in gliomas patients.

## Data Availability

The datasets including the clinical information and the gene expression data of glioma patients analyzed during the current study are available in the TCGA database (https://cancergenome.nih.gov/) and CGGA database (http://www.cgga.org.cn/).
